# Yolov8-CA-EYHL: A bridge crack detection algorithm based on improved YOLOv8

**DOI:** 10.1371/journal.pone.0358872

**Published:** 2026-09-22

**Authors:** Xianwei Zhu, He Chao, Yahui Zhang

**Affiliations:** 1 School of Mechanical and Electrical Engineering, Zhengzhou University of Industrial Technology, Zhengzhou, China; 2 School of Intelligent Manufacturing and Electrical Engineering, Nanyang Normal University, Henan, China; 3 School of Intelligent Manufacturing, Nanyang Institute of Technology, Henan, China; University of Agriculture Faisalabad, PAKISTAN

## Abstract

Automated bridge crack detection is challenging because cracks often exhibit weak contrast, irregular morphology, slender structures, and strong interference from complex surface textures. To address these issues, this study proposes a YOLOv8-CA-EYHL framework that combines filtering-equalization preprocessing with coordinate attention (CA) and enhanced high- and low-frequency feature learning modules. The filtering-equalization strategy improves crack-background contrast while suppressing background interference. CA enhances spatially directional feature representation, while the YHL module strengthens local and high-frequency crack features. The EYHL module further establishes multi-scale feature interaction and spatial recalibration to improve the representation of irregular and slender cracks. Ablation experiments demonstrate that the performance improvement results from the complementary contributions of preprocessing and the proposed architectural components. The proposed model achieves an mAP50 of 93.1% and an mAP50-95 of 63.5%, compared with 64.4% and 42.8%, respectively, for YOLOv8s. It requires 32.6 B FLOPs and 13.7 M parameters while maintaining an inference speed of approximately 76 FPS. Repeated experiments with different random seeds and evaluation on an independent external dataset provide further evidence of the stability and generalisation potential of the proposed framework.

## 1. Introduction

With the rapid development of artificial intelligence [1], the trend of intelligent and unmanned road maintenance has become increasingly prominent [2,3]. Bridges are a critical component of transportation infrastructure safety, and cracks represent one of the most common and potentially hazardous forms of structural deterioration [4,5]. If bridge cracks are not detected and repaired in time, they may continue to propagate, reducing the structure’s load-bearing capacity and durability, and even leading to more serious safety risks [6]. Therefore, efficient and accurate crack detection is essential for bridge health monitoring, maintenance decision-making, and structural safety assessment [7].

Traditional bridge crack inspection primarily relies on manual visual checks. While this method is intuitive and easy to implement, it suffers from low efficiency, strong subjectivity, high labor costs, and significant dependence on lighting conditions, weather, and inspector experience. These limitations make it difficult to meet the demands of large-scale, rapid bridge inspections and intelligent maintenance [8]. Early image processing techniques such as thresholding, edge detection, and texture analysis have been applied to crack identification. However, these methods are highly sensitive to variations in illumination, background textures, and noise, often resulting in false positives or missed detections in complex environments [9]. With the advancement of deep learning, automated crack detection methods based on convolutional neural networks (CNNs) have become a research focus, demonstrating strong feature extraction and generalization capabilities in detecting surface cracks on bridges [7].

In the field of object detection, the YOLO series has gained widespread application in road crack, bridge crack, and surface defect detection due to its end-to-end detection pipeline, fast inference speed, and flexible deployment capabilities [10]. Beyond infrastructure inspection, YOLO-based methods have also been applied to agricultural robotics and plant-related visual recognition. For example, Li et al. developed a root-zone fertilization robot based on YOLOv5 for stalk identification, demonstrating the applicability of YOLO-based object detection in robotic agricultural operations [11]. Yang et al. investigated an SSD-based deep learning framework for automatic road crack detection, improving feature representation by introducing a receptive field module [12]. Zhong et al. proposed a multi-scale feature fusion network for pixel-level pavement damage detection, providing valuable insights into multi-scale crack feature representation [13]. As YOLO models continue to evolve, YOLOv8 has demonstrated outstanding performance in terms of accuracy, inference speed, and architectural flexibility, gradually being adopted for bridge crack and concrete surface defect detection. Xiong et al. introduced a YOLOv8-GAM-Wise-IoU model that integrates global attention mechanisms with an improved loss function, enhancing detection performance on bridge surfaces under complex backgrounds [14]. Su et al. developed an enhanced YOLOv8s model for intelligent asphalt pavement crack detection, demonstrating the effectiveness of YOLOv8 in detecting slender crack targets [15]. Recently, Xia et al. proposed a YOLOv8-based algorithm for bridge crack detection [16]; Xu et al. incorporated improved modules into YOLOv8n to enhance crack detection capability in complex backgrounds [17]; Li et al. combined attention mechanisms with deformable convolutions to improve detection performance for surface defects on concrete bridges [18]. Liu et al. proposed the FEGW-YOLO framework, enabling lightweight networks to maintain multimodal input capabilities while supporting real-time perception and operational precision required for autonomous operations [19]. Prabu et al. constructed a night-vision detection framework that combines 3D spatiotemporal modeling with a handcrafted-inspired parameter search strategy, addressing challenges in identifying weak features and disordered lighting in low-light images [20]. Zhang et al. proposed AL-YOLOv8, which employs an adaptive spatial feature fusion detection head, large-kernel separable attention mechanisms, and a loss function integrating dynamic attention with InnerIoU, effectively solving issues such as background interference, insufficient receptive fields, and regression bias in remote sensing small-object detection [21].

Although existing YOLO-based models have achieved promising results in crack detection, bridge cracks remain challenging because of their slender structures, large scale variations, irregular contours, and complex textured backgrounds. Fine and weakly textured cracks may lose detailed features during feature extraction, while insufficient multi-scale feature interaction can lead to missed detections and inaccurate localization [22]. Existing studies have demonstrated that attention mechanisms and multi-scale feature fusion can improve YOLOv8-based detection in complex scenes [23]. However, the joint representation of directional information, high-frequency crack details, and multi-scale spatial features remains insufficient for complex bridge surfaces.

To address these limitations, this study proposes an improved YOLOv8-CA-EYHL framework for bridge crack detection. The main contributions are as follows:

(1) A dual-stage coordinate attention strategy is introduced into the backbone and neck of YOLOv8 to enhance spatially directional feature representation during feature extraction and multi-scale feature fusion.
(2) The YHL module is incorporated into C2f to strengthen high-frequency and local texture feature extraction, thereby improving the representation of slender and weakly textured cracks.
(3) An EYHL module is designed to enhance multi-scale spatial interaction and feature recalibration between shallow and deep features, improving the detection of cracks with different scales and morphological characteristics.

## 2. Materials and methods

### 2.1. Datasets

To validate the effectiveness of the proposed bridge crack detection model, a dataset of bridge crack images was constructed. These images were collected from publicly accessible bridge surface scenarios under various real-world environmental conditions, covering different lighting conditions, shooting angles, background textures, and crack patterns. Therefore, the constructed dataset can reasonably reflect the complex environmental characteristics encountered in practical bridge crack detection tasks. Based on crack morphology and engineering inspection requirements, bridge cracks were categorized into four types: transverse cracks, longitudinal cracks, large obvious cracks, and irregular cracks. The categories and their corresponding label codes are shown in Table 1.

**Table 1:** Classification and label encoding of bridge cracks.

Type	Feature code	Label
**Transverse crack**	T00	T
**Longitudinal crack**	L00	L
**Large obvious crack**	R00	R
**Irregular crack**	I00	I

The bridge crack dataset in the experimental setup contains 5,200 annotated images, including 1,300 horizontal crack images, 1,300 longitudinal crack images, 1,300 large and obvious crack images, and 1,300 irregular crack images. The dataset is randomly divided into training, validation, and test subsets in a 70%:15%:15% ratio, comprising 3,640 training images, 780 validation images, and 780 test images. All comparative and ablation experiments adopt the same partitioning scheme to ensure fair evaluation.

Field Site Access and Permits. The bridge crack images used in this study were collected from publicly accessible bridge structures for research and algorithm validation purposes. No specific permits were required for the image acquisition because the data collection activities did not involve protected areas, private properties, human participants, or restricted infrastructure operations. The images were obtained without affecting normal bridge usage or maintenance activities.

**Fig 1:**
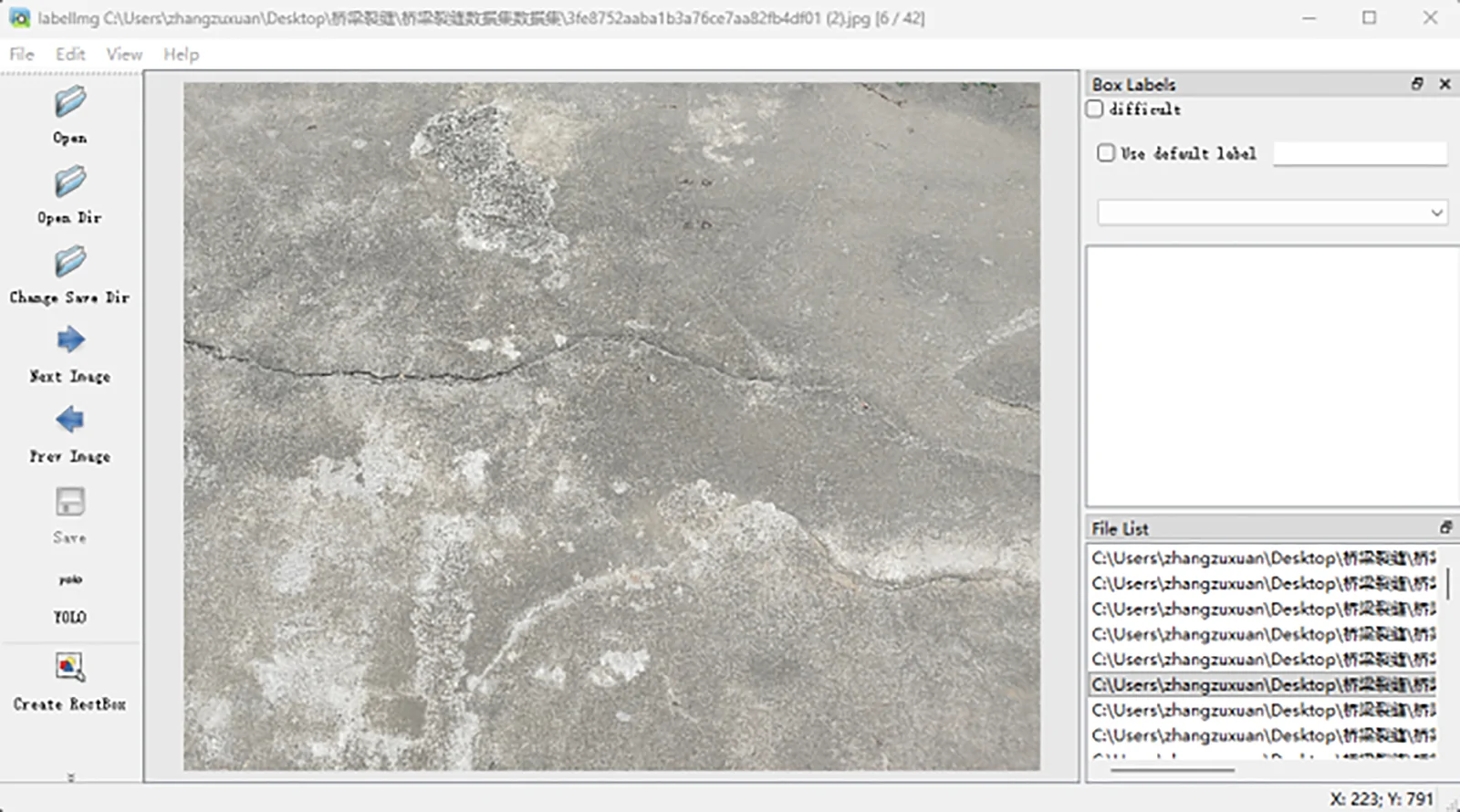
Annotation examples of bridge crack images generated by the authors using LabelImg.

**Fig 2:**
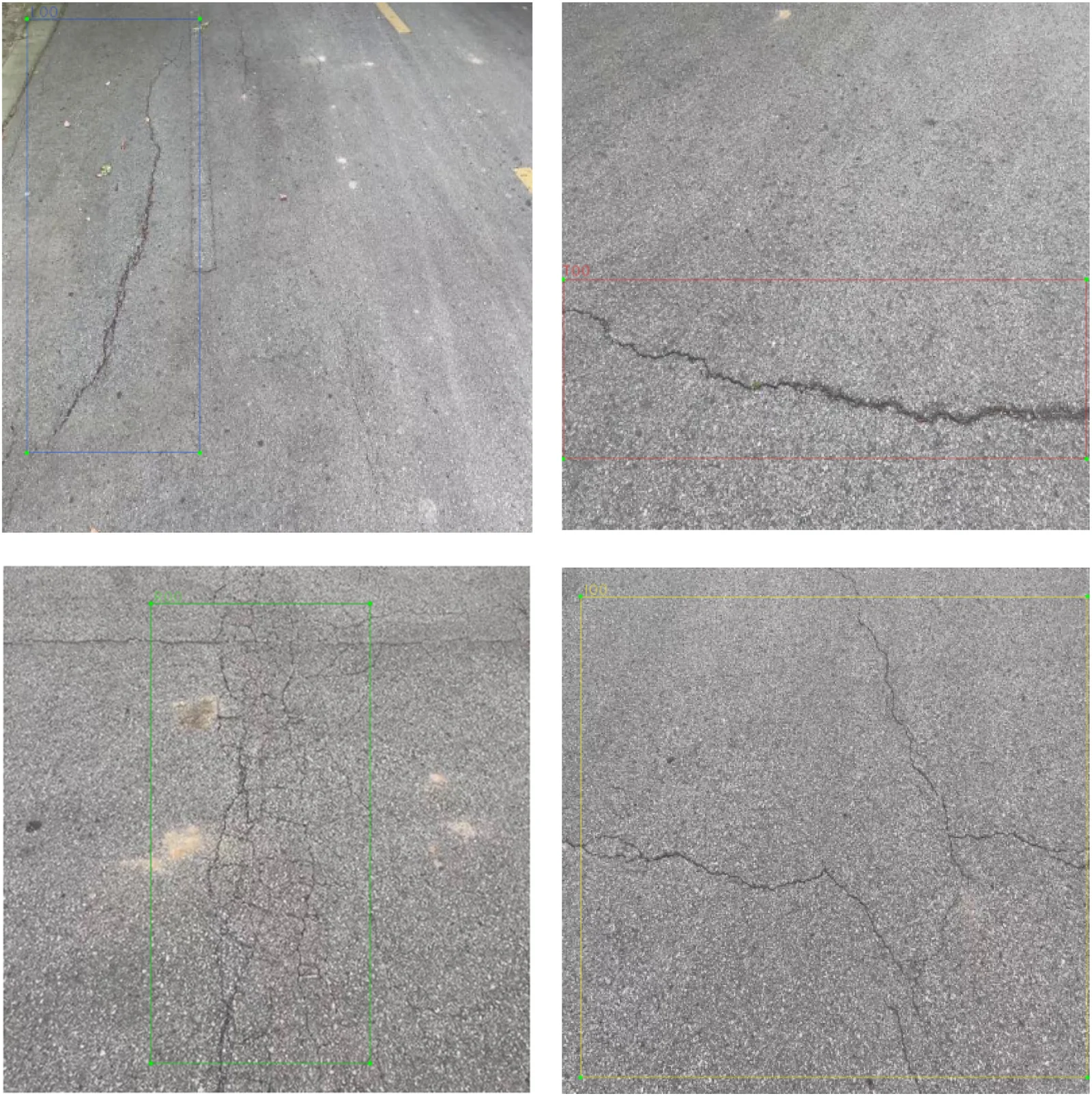
Representative bridge crack images from the constructed dataset.

Feature codes are used to distinguish crack images of different categories, while labels serve for category annotation during model training. Crack targets were manually annotated using LabelImg, with annotation formats conforming to the requirements of the YOLO object detection framework. To ensure data quality, crack boundaries were carefully delineated as accurately as possible, and images with ambiguity, occlusion, or unclear boundaries were manually reviewed. The annotation examples generated by the authors are shown in Fig 1, and representative bridge crack images from the four categories in the constructed dataset are presented in Fig 2.

Bridge cracks are usually related to material aging, structural deformation under load, temperature changes, construction defects, and external environmental erosion. Different types of cracks have significant differences in morphology, length, width, and direction. Longitudinal cracks generally extend along the longitudinal direction of the bridge structure, transverse cracks are often associated with structural loads and thermal stress, obvious large cracks have high visibility and wider width, and irregular cracks present complex shapes and variable directions. These differences place high demands on the model’s feature extraction and multi-scale detection capabilities.

### 2.2. Image Preprocessing

Bridge crack images are easily affected by illumination changes, shadow occlusion, complex background textures, and noise during the acquisition process. These factors can reduce the contrast between the crack area and the background, thereby affecting the extraction of crack edges and detailed features. Therefore, performing appropriate image preprocessing before model training can improve the distinguishability of cracks and enhance the quality of training data.

#### 2.2.1. *Histogram equalization.*

Histogram equalization is a commonly used image enhancement method. Its basic principle is to redistribute the gray values of the image to make the gray distribution more uniform, thereby improving the overall image contrast. For an input grayscale image, its gray value is $r_{k}\;$, the total number of gray levels is L, and the probability of gray value $r_{k}$ can be expressed as:


$$\begin{array}{c} \begin{array}{c} p(r_{k})=\frac{n_{k}}{MN},\; \end{array}k=\text{0},\text{1},\ldots ,L-\text{1} \end{array}$$ (1)

Where $n_{k}$ is the number of pixels corresponding to the gray value $r_{k}$, M and N are the number of rows and columns of the image respectively. The cumulative distribution function of the gray value $r_{k}$ can be expressed as:


$$\begin{array}{c} T(r_{k})=sum_{j=\text{0}}^{k}p(r_{j}) \end{array}$$ (2)

Then, the original gray value is mapped to a new gray value through the cumulative distribution function, that is, histogram equalization is achieved:


$$\begin{array}{c} s_{k}=(L-\text{1})T(r_{k}) \end{array}$$ (3)

where $s_{k}$represents the gray value after equalization. When the gray range is [0,255], the mapping relationship can be correspondingly expressed as: Through this gray mapping, the areas with concentrated gray distribution will be stretched, thereby improving the overall contrast of the image. For bridge crack images, histogram equalization can enhance the gray difference between the crack area and the background, making the crack edges more clearly visible.

However, traditional histogram equalization is a global enhancement method that fails to fully consider local texture differences or noise distribution. When the original image contains complex background textures, uneven illumination, or noise interference, global histogram equalization may simultaneously amplify background noise, causing crack details to be disturbed by irrelevant textures. Therefore, relying solely on traditional histogram equalization cannot stably improve crack detection performance in complex bridge surface images.

#### 2.2.2. *Filtering-equalization enhancement algorithm.*

**Fig 3:**
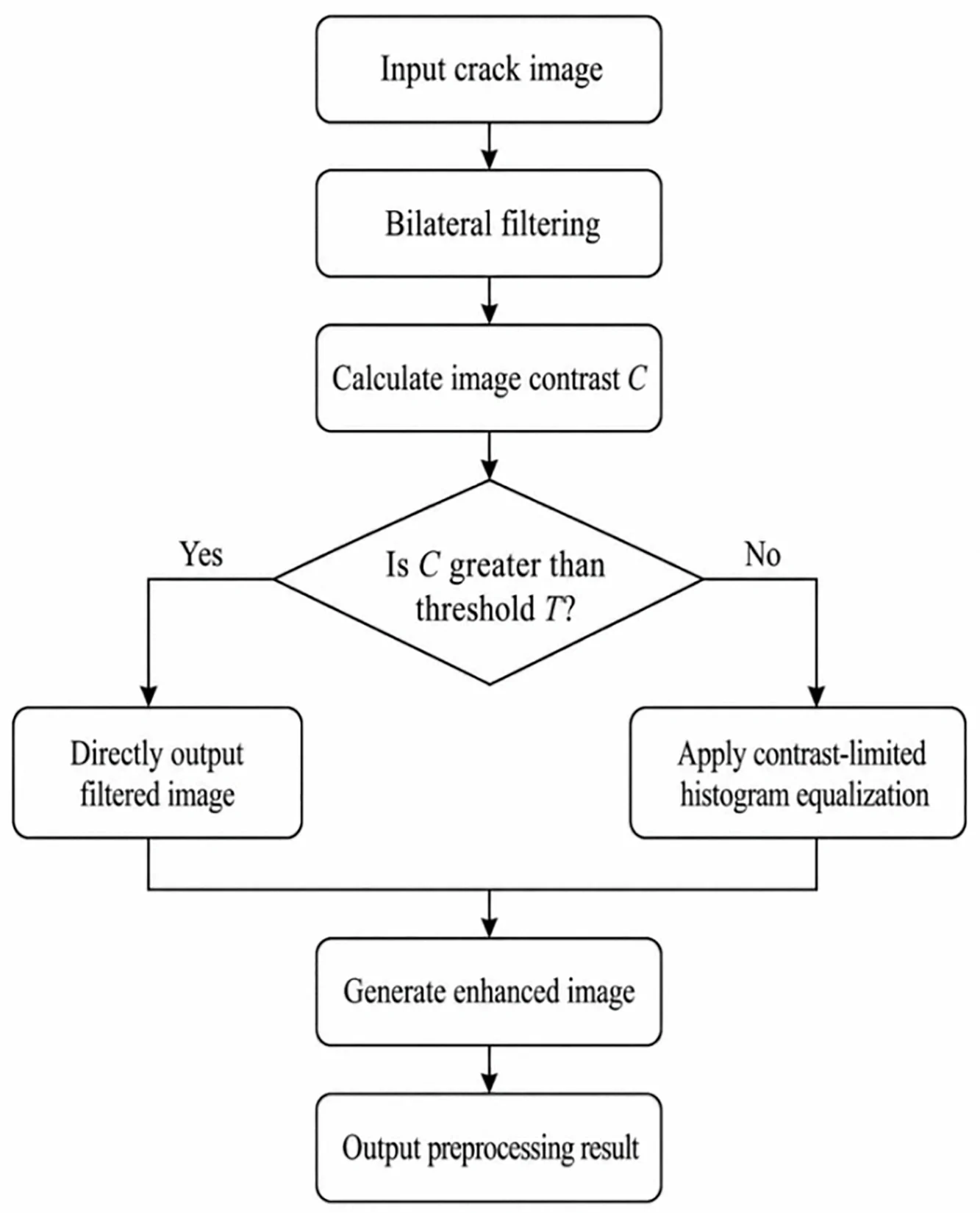
Flowchart of the Filtering-Equalization Enhancement Algorithm.

To address the problem that traditional histogram equalization tends to amplify noise and weaken crack detail recognition, this paper adopts a filtering-equalization enhancement algorithm to preprocess crack images. This algorithm first smooths the input image through bilateral filtering to reduce background noise while preserving crack edge information as much as possible. Then, the image contrast is calculated, and based on a preset threshold, it is determined whether contrast-limited histogram equalization should be further applied. In this study, the contrast threshold is set to T = 1.8, the spatial distance parameter of bilateral filtering is set to 4, and both the color space and coordinate space parameters are set to 80. Let the contrast of the filtered image be C. If C meets the preset threshold, the contrast is considered sufficient to extract crack features, and the filtered image is directly output; otherwise, if the contrast between the crack area and the background is still insufficient, contrast-limited histogram equalization is further applied. The algorithm flow is shown in Fig 3.

Compared with the traditional histogram equalization method, the filtering-equalization enhancement algorithm can suppress background noise while enhancing the contrast of the crack area, and avoid excessive enhancement of overall brightness and texture. Therefore, this method is more suitable for the preprocessing of bridge crack images with complex backgrounds.

#### 2.2.3. *Comparative analysis of preprocessing effects.*

**Figure 4:**
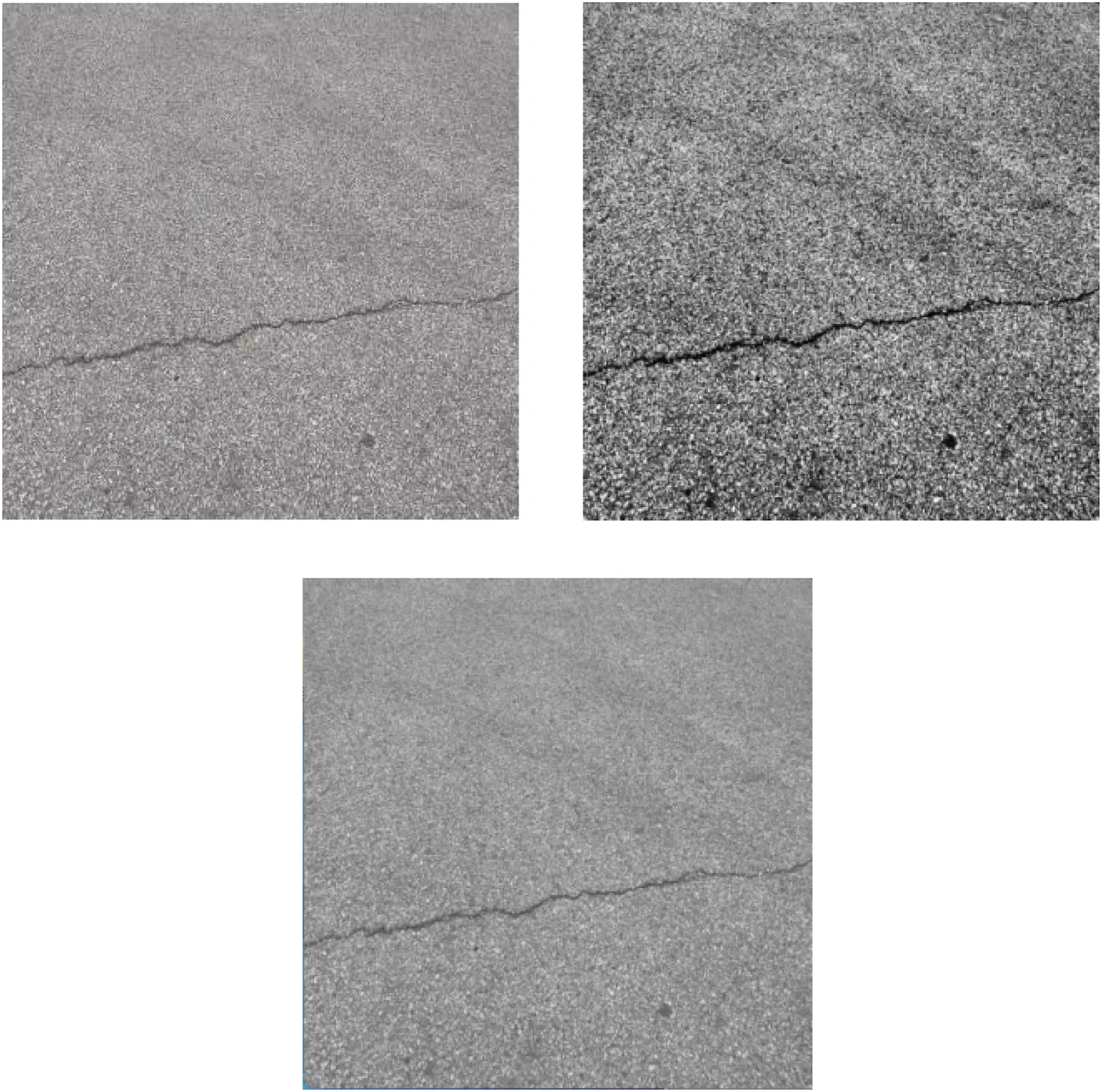
Architecture of the proposed YOLOv8-CA-EYHL framework. (Source: Figure created by the authors.).

To qualitatively evaluate the effectiveness of the proposed preprocessing strategy, the original image, histogram-equalized image, and filtering-equalization enhanced image are compared using a representative transverse crack image, as shown in Fig 4.

As shown in Fig 4, the original image exhibits relatively low contrast between the crack and surrounding background. Traditional histogram equalization improves overall contrast but also enhances background texture and noise. In comparison, the filtering-equalization strategy suppresses background interference while preserving and enhancing crack edge information. These results indicate that the proposed preprocessing method can provide clearer crack-related features for subsequent detection.

## 3. Methods

### 3.1. Structural framework

**Fig 5:**
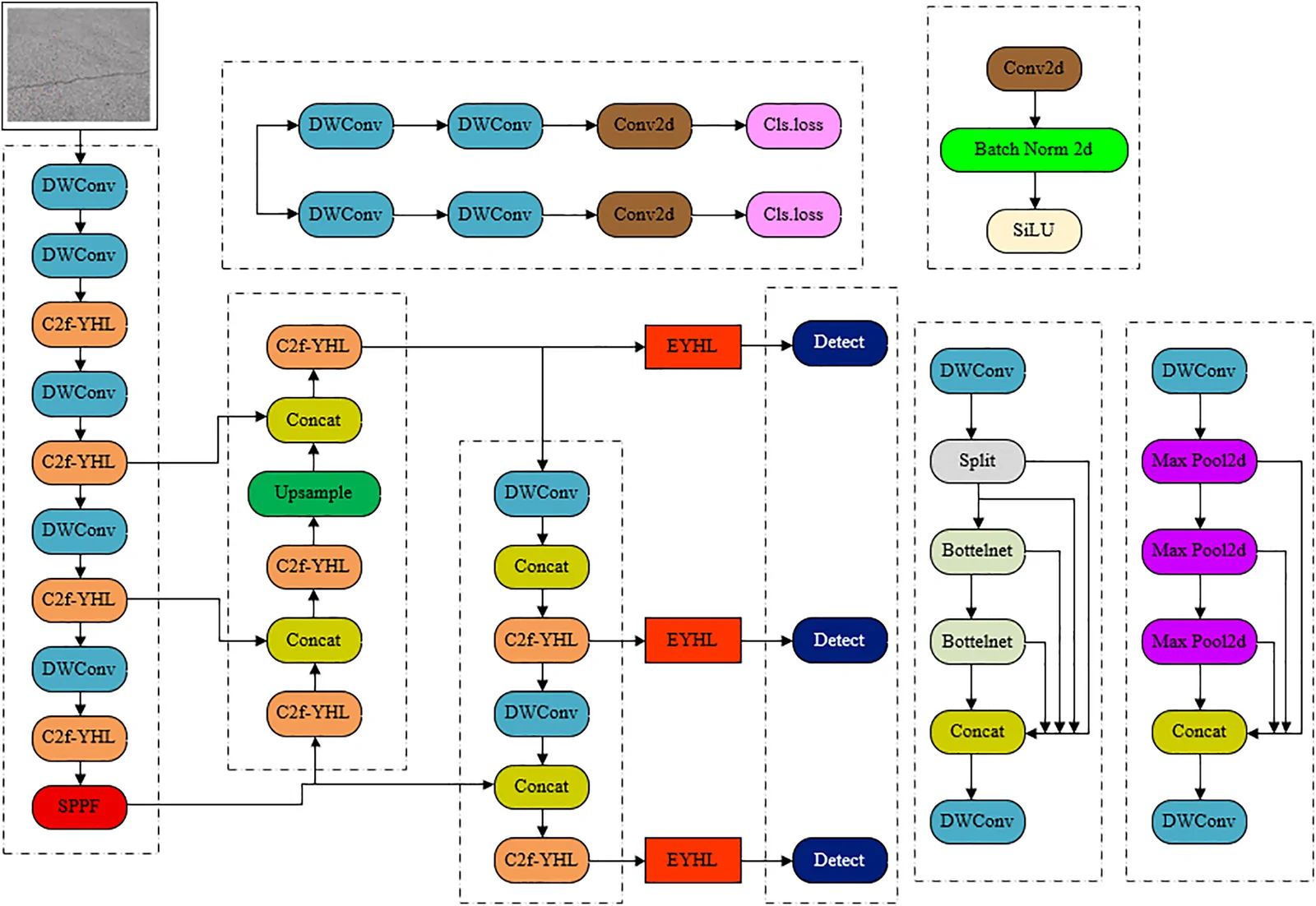
Improved YOLOv8-CA-EYHL network architecture.

To address the problems of slender bridge crack targets, inconspicuous features of tiny cracks, and false detections or missed detections under complex backgrounds, this paper proposes an improved YOLOv8-CA-EYHL bridge crack detection algorithm. The overall architecture of the proposed model is illustrated in Fig 5. First, the CA mechanism is added to the backbone network and neck network of YOLOv8 to optimize the multi-scale feature fusion effect. Secondly, the YHL module is used to replace the BottleNeck basic unit with feature extraction redundancy inside C2f, so as to strengthen the network’s ability to capture image features. Finally, to address the problem of insufficient multi-scale target recognition performance in the original YOLOv8 bridge crack detection task, a multi-scale spatial recalibration network EYHL is designed to enhance the model’s multi-scale feature capture capability. The combination of these components is intended to enhance crack feature representation, improve multi-scale feature fusion, and reduce interference from complex backgrounds. The individual contributions of these components are further evaluated through ablation experiments in Section 4.5.

### 3.2. Coordinate attention principle

**Fig 6:**
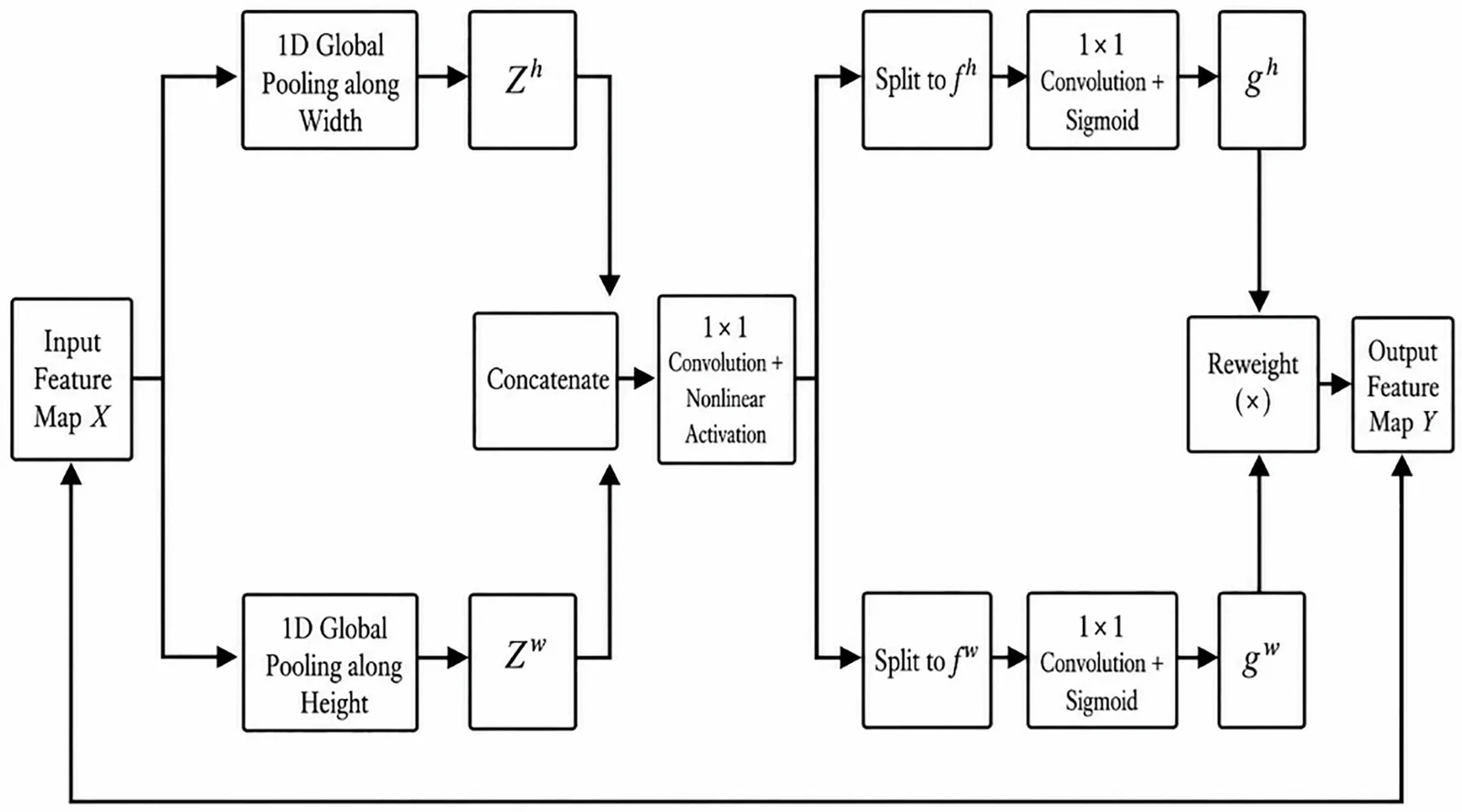
Schematic diagram of the coordinate attention mechanism.

Coordinate attention was proposed by Hou, which embeds spatial coordinate information into channel attention. Its core idea is to enable the model not only to identify which channels are important, but also to perceive the position of target features in the image. Unlike traditional channel attention, CA performs one-dimensional global pooling in the horizontal and vertical directions, retaining positional information when generating attention weights, thereby improving the modeling capability for direction-aware features and long-range dependencies. The structure of the CA module is shown in Fig 6.

Coordinate Attention PrincipleLet the input feature map be:


$$\begin{array}{c} X\in R^{C\times H\times W} \end{array}$$ (4)

where C, H, and W represent the number of channels, height, and width of the feature map respectively. Traditional channel attention usually compresses each channel into a scalar through two-dimensional global pooling, which may lead to the loss of spatial position information. CA decomposes the two-dimensional global pooling into two one-dimensional global pooling operations along the horizontal direction and the vertical direction.

For the c-th channel, the feature aggregation along the horizontal direction can be expressed as:


$$\begin{array}{c} z_{c}^{h}\left(h\right)=\frac{1}{W}\sum 0\leq i<Wx_{c}\left(h,i\right) \end{array}$$ (5)

where $z_{c}^{h}\left(h\right)$ represents the aggregated feature at height *h* in the *c* th channel, and *W* is the width of the feature map.

The feature aggregation along the vertical direction can be expressed as:


$$\begin{array}{c} z_{c}^{w}\left(w\right)=\frac{1}{H}\sum 0\leq j<Hx_{c}\left(j,w\right) \end{array}$$ (6)

where $z_{c}^{w}(w)$ represents the aggregated feature at width w in the c-th channel, and H is the height of the feature map. The feature maps obtained from the two directions are then concatenated and passed through a 1 × 1 convolutional layer to reduce the channel dimension, thereby obtaining an intermediate feature representation. This operation enhances the feature representation capability while reducing computational cost. The intermediate feature is then split into horizontal and vertical components, which are respectively processed by two independent convolutional layers and Sigmoid activation functions to generate attention weights for different directions. Finally, the attention weights from the two directions are element-wise multiplied with the original input feature map to obtain the enhanced output feature map. Through this process, the CA module can enhance the response of key crack areas while suppressing background noise and irrelevant texture interference.

When the CA module is embedded into YOLOv8, the model can more accurately capture the directional extension information and spatial position information of crack targets. Since bridge cracks usually appear as slender, continuous, and directional patterns, CA can effectively improve the model’s ability to recognize tiny cracks, irregular cracks, and cracks in complex backgrounds, thereby alleviating the problems of missed detections and false detections in the original YOLOv8 model.

### 3.3. YHL and EYHL feature enhancement modules

#### 3.3.1 *C2f Enhancement module.*

The C2f module in YOLOv8 improves feature representation through cross-stage feature fusion. However, repeated convolution and downsampling may weaken fine crack textures, particularly for slender and weakly textured targets. To address this problem, the YHL module is introduced to replace the Bottleneck units within C2f and strengthen high- and low-frequency feature representation.

**Fig 7:**
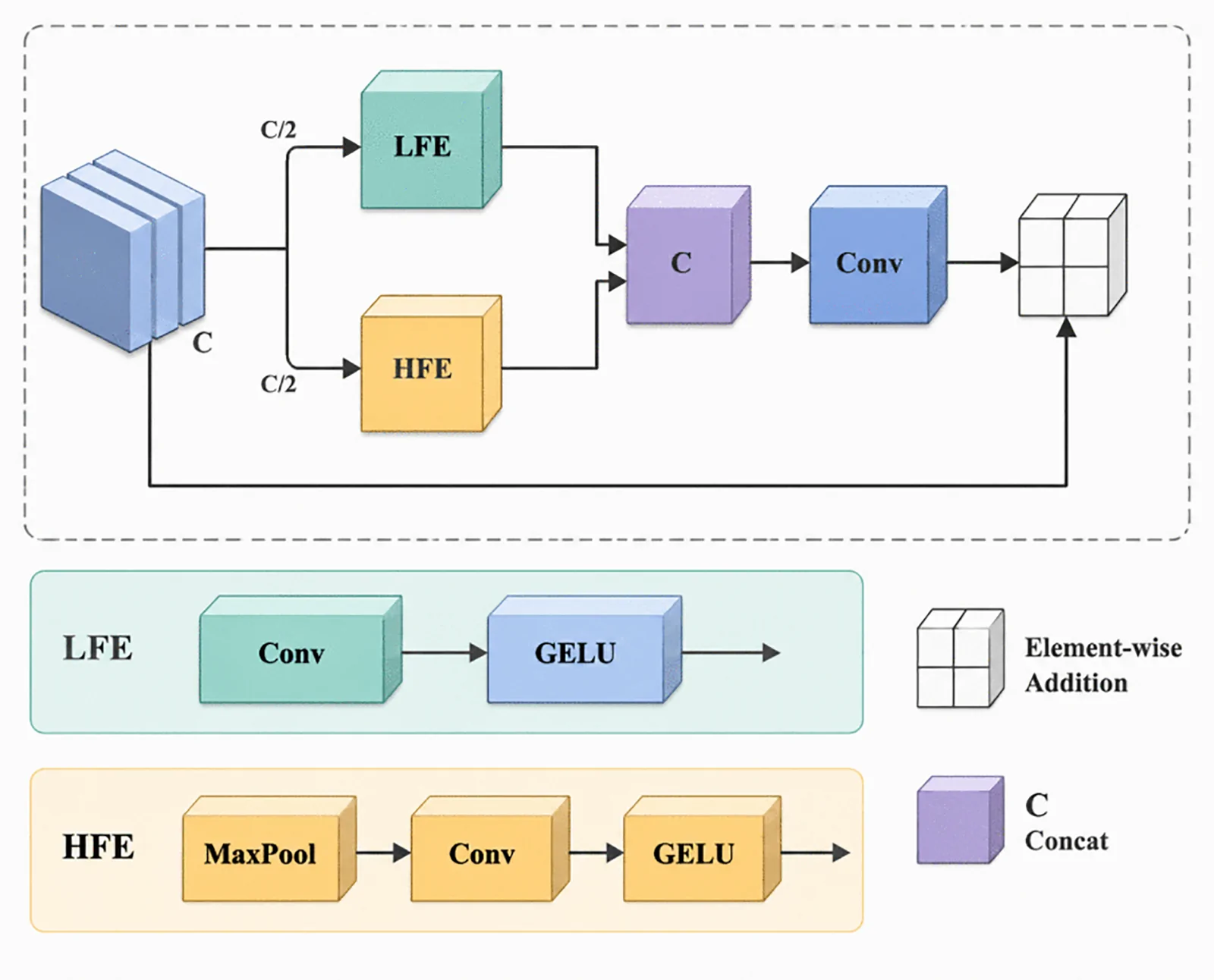
YHL Structure.


The YHL module consists of high-frequency enhancement (HFE) and low-frequency enhancement (LFE) branches, as shown in Fig 7. HFE focuses on edge and fine-texture information, whereas LFE captures local contextual features. Their complementary operation improves the representation of crack boundaries and local textures. The detailed operations are defined by Eqs. (7)-(9).

$$\begin{array}{c} X=C_{1\times 1}\left(\left[{\hat{F}}_{in}^{LFE},{\hat{F}}_{in}^{HFE}\right]\right)+F_{in} \end{array}$$ (7)

$$\begin{array}{c} {\tilde{F}}_{in}^{HFE}=g_{a}\left(C_{1\times 1}\left(P_{𝐌𝐚𝐱}\left({\tilde{F}}_{in}^{HFE}\right)\right)\right) \end{array}$$ (8)

$$\begin{array}{c} {\tilde{F}}_{in}^{LFE}=g_{a}\left(C_{3\times 3}\left({\tilde{F}}_{in}^{LFE}\right)\right) \end{array}$$ (9)

Where ${\hat{F}}_{in}^{HFE}\;$HFE is the input of HFE, ${\hat{F}}_{in}^{LFE}$LFE is the input of LFE, $C_{\text{k}\times \text{k}}$ refers to the convolution size, $g_{a}$refers to the GELU activation function, $P_{\text{Max}}$represents max pooling, and [,] represents the concatenation operation.

#### 3.3.2. *EYHL multi-scale spatial enhancement.*

To further improve the representation of cracks with different scales and morphological characteristics, an enhanced YHL (EYHL) module is designed. The module integrates four sequential operations: multi-scale feature fusion, spatial selection, spatial interaction, and feature recalibration.

The HFE sub-module inside the YHL module is mainly used to strengthen high-frequency features of the image, that is, detailed texture information such as object contours and edge boundaries. This branch first retains image detail features through max pooling operation, and then performs channel dimension fusion on the pooled features with the help of 1 × 1 convolution, without changing the spatial size of the feature map throughout the entire process; finally, the GELU activation function is introduced to further highlight the feature weights of high-frequency textures. In contrast, the LFE sub-module relies on local convolution operations to capture subtle features within the region and complete detail information extraction. After the convolution operation output is activated by GELU, a layer of nonlinear transformation is also superimposed to further optimize the network feature representation effect.

**Fig 8:**
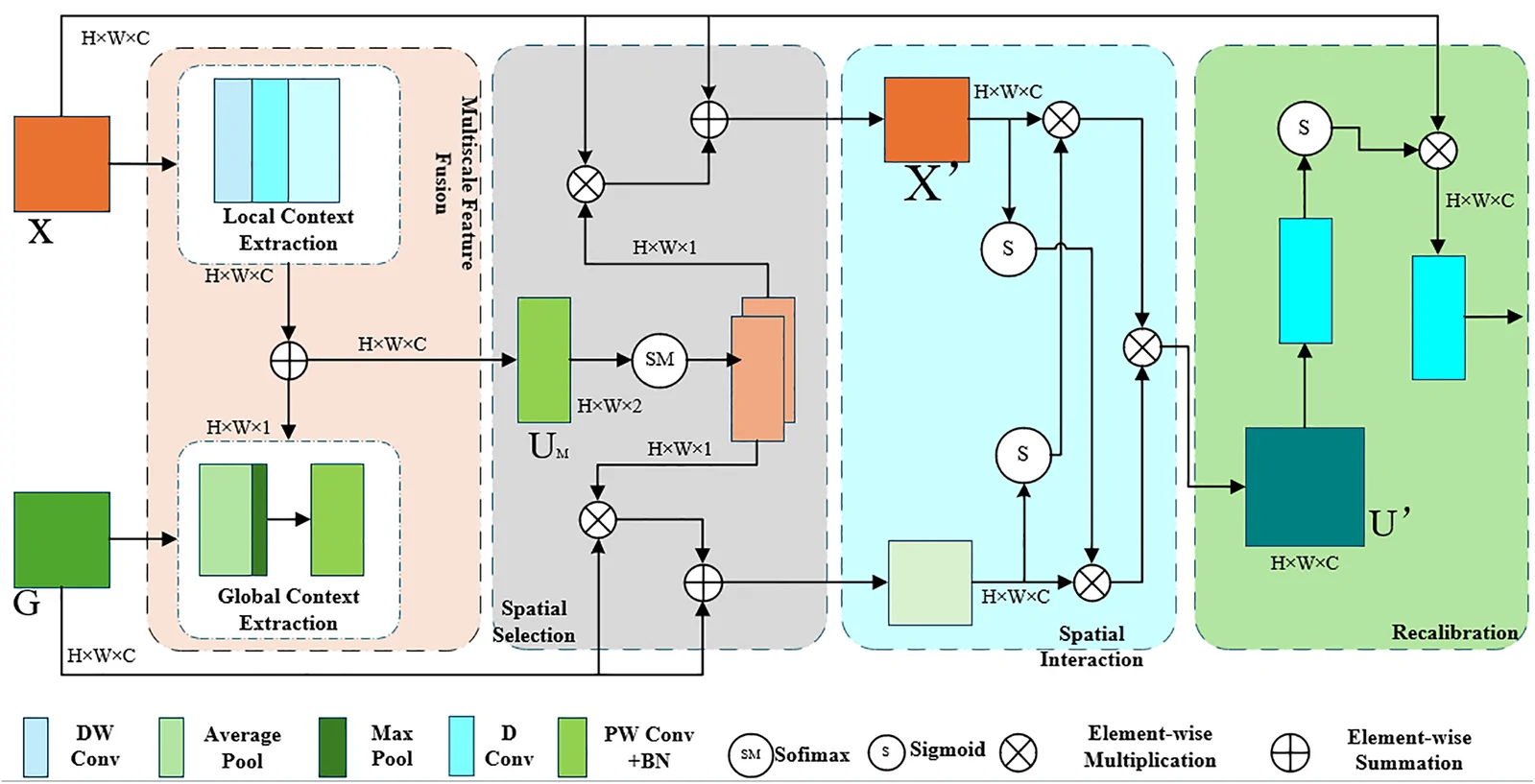
EYHL Structure Diagram.

To address the problem of insufficient multi-scale target recognition performance in the original YOLOv8 bridge crack detection task, this paper designs a multi-scale spatial recalibration network EYHL to enhance the model’s multi-scale feature capture capability. The overall structure of the network is shown in Fig 8.

The network is divided into four major processing flows, which sequentially complete multi-scale feature fusion, spatial region selection, spatial feature interaction, and feature recalibration operations.

In the multi-scale fusion stage, the shallow feature X is first processed: 3 × 3 depthwise convolution and dilated convolution are sequentially used to expand the receptive field, and then 1 × 1 pointwise convolution is used to unify the feature channel dimension, so that the number of channels is consistent with that of the global branch. This processing scheme can preserve the original high-resolution advantage of shallow features while broadening the network’s receptive field, making it easier for the model to extract fine local textures within the image.

For the deep feature G, two operations of average pooling and max pooling are performed in parallel, and then a deep feature map with strong semantic information is generated with the help of 1 × 1 pointwise convolution. After completing the respective transformations of shallow and deep features, the two types of features are fused by element-wise addition to obtain a multi-scale fused feature map.

The core role of this stage is to integrate shallow local details and deep global context information, allowing the network to fully utilize large-scale environmental semantics while extracting fine-grained target features. This fusion approach can adapt to detection targets with large size differences, taking into account both local texture and global environmental features, effectively improving multi-scale target recognition accuracy. The improvement in detection performance is particularly evident in dynamic and complex scenes. The complete operation process of this stage is shown in Equation (10).


$$\begin{array}{c} U_{M}=C_{1\times 1}(D(D_{W}(X)))+C_{1\times 1}([\begin{array}{c} P_{A\nu g}(G),P_{Max}\left(G\right) \end{array}]) \end{array}$$ (10)

Where $U_{M}$ represents the feature map after multi-scale fusion, $D_{W}$ and *D* represent depthwise convolution and dilated convolution respectively. Ckxk represents k × k convolution, $P_{A\nu g}$ and $P_{Max}$ represent average pooling and max pooling respectively, and [·] represents the concatenation operation.

In the spatial selection stage, a 1 × 1 convolution operation is first performed on the fused feature map U, and then a spatial weight distribution map adapted to shallow and deep features is generated through the softmax function. The obtained weight maps are respectively subjected to weighted residual fusion with the original shallow feature X and deep feature G, and finally the weighted and optimized features X′ and G′ are output. The softmax function can assign corresponding weights according to the importance of the target area within the image, strengthen the feature response of the key target area, and simultaneously weaken the interference caused by irrelevant background areas. After this spatial weight selection and optimization, the representation effect of the feature map is significantly improved, and the accuracy of the model in recognizing targets is also enhanced. The relevant operation process of this stage is given by Equations (11) to (12).


$$\begin{array}{c} X^{\prime }=S(C_{1\times 1}(U_{M}))⊗X+X \end{array}$$ (11)

$$\begin{array}{c} G^{\prime }=S(C_{1\times 1}(U_{M}))⊗G+G \end{array}$$ (12)

Where X′ and G′ represent the feature maps after weighted residual fusion, S() represents the softmax operation, and ⊗ represents element-wise multiplication.

In the spatial interaction stage, the weighted fused feature maps X′ and G′ are further optimized. First, the two sets of feature maps are input into the sigmoid activation function to generate the corresponding spatial attention weight matrices, so as to achieve complementary weighted optimization of shallow and deep features. In the specific operation, the attention weights obtained through sigmoid activation are element-wise multiplied with the original input feature maps of this stage to generate brand-new weighted feature maps. On this basis, the two types of weighted feature maps undergo bidirectional interactive fusion, allowing shallow detail features and deep semantic features to empower each other and achieve complementary optimization. This bidirectional feature interaction mechanism can effectively guide the model to focus on the core target area, weaken invalid feature interference, and significantly improve the target detection accuracy of the algorithm. The specific calculation method of this stage is shown in Formula (13).


$$\begin{array}{c} U^{\prime }=(X^{\prime }⊗\sigma (G^{\prime }))⊗(G^{\prime }⊗\sigma (X^{\prime }))\; \end{array}$$ (13)

Where U′ represents the feature map after spatial cross-domain fusion, and σ() represents the Sigmoid activation function.

Recalibration stage. First, 1 × 1 convolution dimensionality reduction and feature integration operations are performed on the interacted feature map U′, combined with batch normalization to correct the feature distribution, and then the final spatial attention feature map is generated through the sigmoid activation function. This network operation can assign a specific weight coefficient to each spatial position of the feature map, quantifying the contribution degree of different regions to the target detection task. The generated weight matrix is weighted and multiplied with the shallow feature map to complete the secondary calibration optimization of global features. This operation can significantly enhance the feature response capability of the target core area and suppress the interference noise caused by complex backgrounds. The feature map after recalibration processing can effectively highlight the effective features of the target and eliminate irrelevant background information, providing high-quality feature input for the subsequent detection network. Ultimately, the detection accuracy and generalization robustness of the model in various scenarios are both improved. The calculation process is as follows:


$$\begin{array}{c} X=C_{1\times 1}(\sigma (C_{1\times 1}(U^{\prime }))⊗X) \end{array}$$ (14)

**Fig 9:**
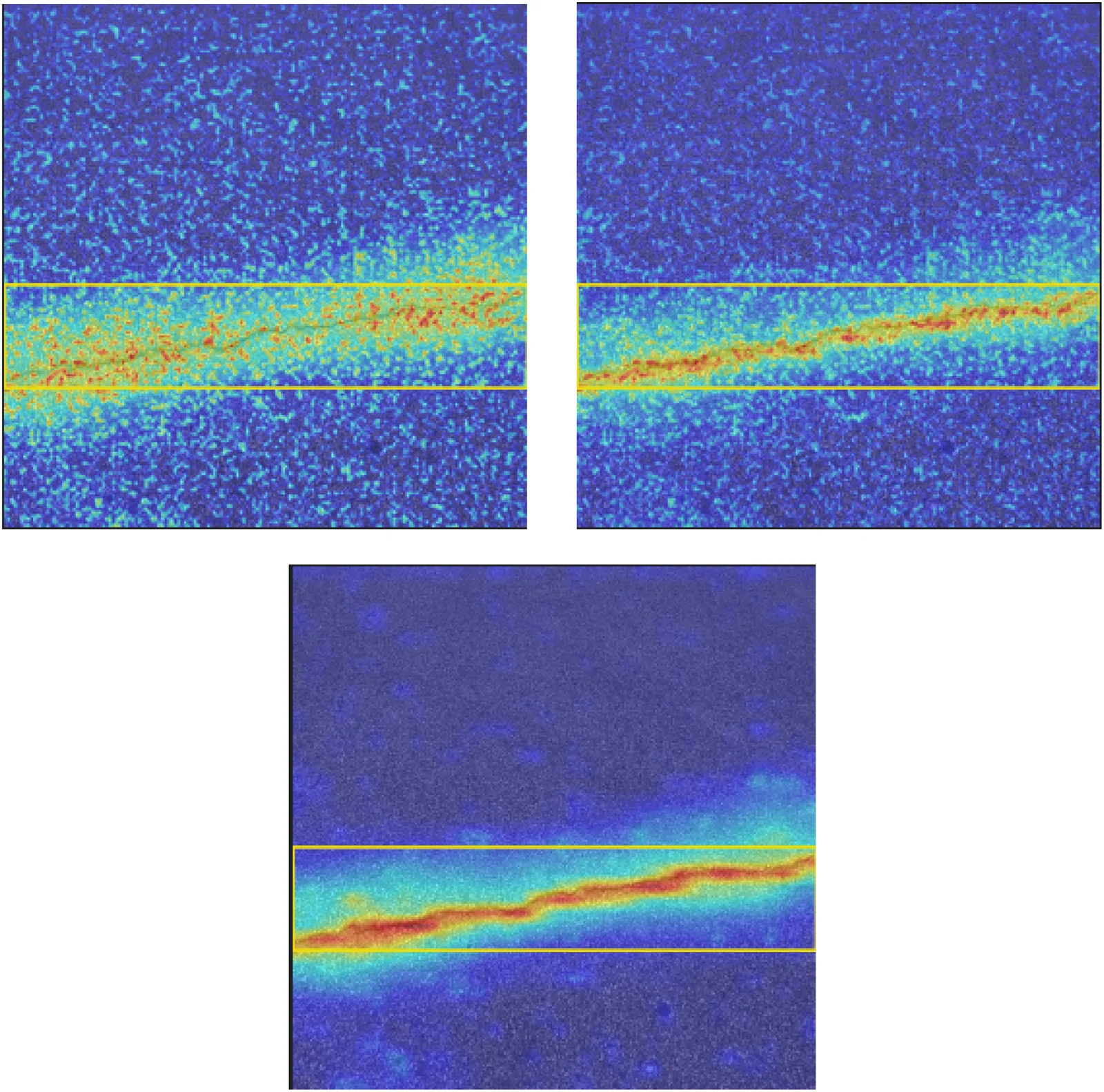
Compares the feature activation maps of the baseline YOLOv8s and the proposed YOLOv8-CA-EYHL model.

The improvement of the improved model in feature extraction capability compared with the original model is shown in Fig 9.

## 4. Experimental results and analysis

### 4.1. Experimental environment and parameter settings

To verify the effectiveness of the proposed YOLOv8-CA-EYHL bridge crack detection model, model training, comparative experiments, and ablation experiments were conducted on the constructed bridge crack dataset. The hardware configuration is as follows: Intel(R) Core(TM) i7-12800HX CPU, main frequency 4.8 GHz, 32 GB memory, NVIDIA GeForce RTX 4060Ti GPU. This hardware platform can meet the training and inference requirements of the YOLO-based bridge crack detection model.

**Fig 10:**
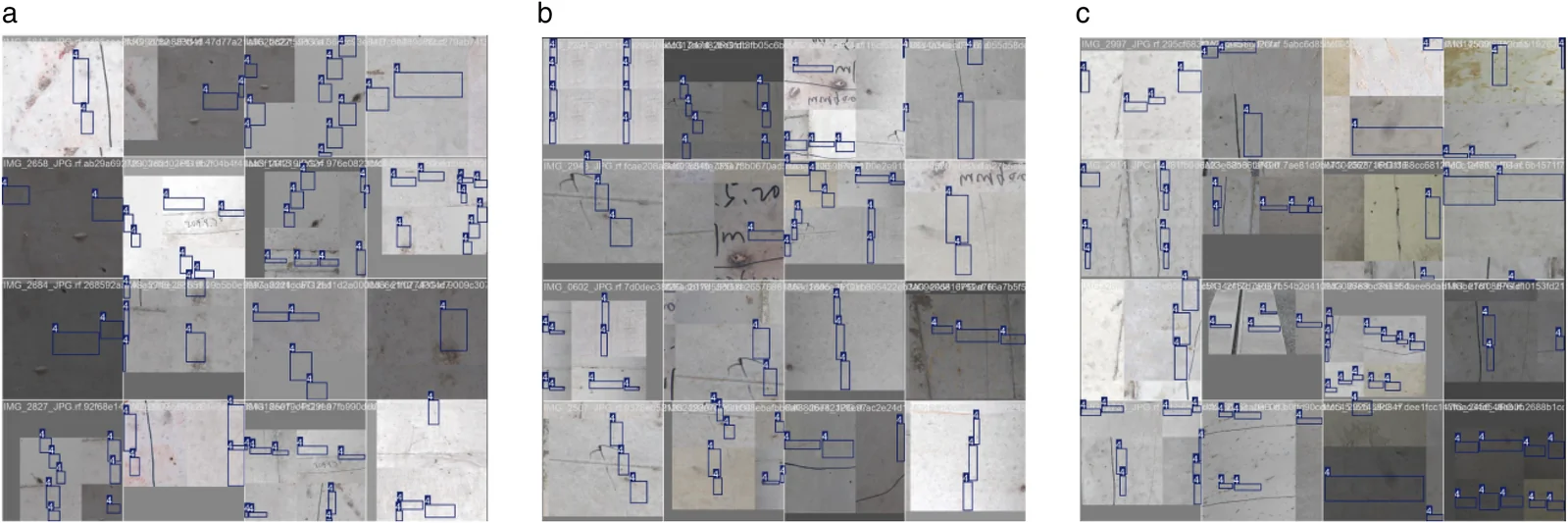
Visualization of training samples. (a) train_batch0, (b) train_batch1, (c) train_batch2.

In terms of the software environment, a virtual environment was created using Anaconda, and Python 3.10 was used in PyCharm for model training and testing. During the training process, before inputting images into the model, data augmentation operations including rotation, scaling, and cropping were applied to enhance the model’s adaptability to different shooting angles, scale variations, and complex background conditions. Fig 10 shows the visualization results of the training samples.

The number of training iterations was set to 100, the batch size was set to 32, and the optimizer used stochastic gradient descent (SGD). Since filtering-equalization enhancement had already been performed in the data preprocessing stage, the Mosaic augmentation parameter was set to 0 to avoid excessive data perturbation affecting crack detail features. The training parameters are listed in Table 2.

**Table 2:** Model training parameter settings.

Parameter	Setting
**Initial learning rate (lr0)**	**0.01**
**Final learning rate (lrf)**	**0.02**
**Batch size**	**32**
**Weight decay**	**0.001**
**Warmup epochs**	**3.0**
**Epochs**	**100**
**Optimizer**	**SGD**
**Mosaic augmentation**	**0.0**

To ensure a fair comparison, all comparison models were trained using the same training dataset division, training epochs, and main hyperparameter settings. YOLOv5s, YOLOv8n, YOLOv8s, YOLO11s, YOLOv8-SE, and YOLOv8-CBAM were selected as comparison models to comprehensively evaluate the performance of the proposed YOLOv8-CA-EYHL model in terms of model complexity, detection accuracy, and inference speed.

### 4.2. Evaluation metrics

To comprehensively evaluate the performance of the improved model in bridge crack detection, commonly used object detection evaluation metrics were adopted, including precision, recall, F1 score, mean average precision (mAP50 and mAP50-95), and inference speed (FPS).

Precision represents the proportion of samples predicted as cracks that are actually cracks, and is used to measure false detection conditions:


$$\begin{array}{c} \text{Precision}=\frac{TP}{TP+FP} \end{array}$$ (15)

Where TP represents the number of cracks truly detected, and FP represents the number of falsely reported cracks. The higher the precision, the lower the false detection rate.


$$\begin{array}{c} \text{Recall}=\frac{TP}{TP+FN} \end{array}$$ (16)

Where FN represents the number of real crack targets that the model fails to detect. The higher the recall, the lower the missed detection rate.

F1 score is the harmonic mean of precision and recall, which can comprehensively reflect detection performance:


$$\begin{array}{c} F1-\text{score}=2\times \frac{\text{Precision}\times \text{Recall}}{\text{Precision}+\text{Recall}} \end{array}$$ (17)

Mean average precision (mAP) is a comprehensive metric widely used in object detection. mAP50 represents the mean average precision when the intersection over union (IoU) threshold is 0.5, which is mainly used to evaluate the basic target detection capability. mAP50-95 represents the average mAP calculated over the range of IoU thresholds from 0.5 to 0.95 (with a step size of 0.05), which is more stringent in evaluating localization accuracy. IoU is calculated as the ratio of the intersection area to the union area between the predicted bounding box and the ground truth bounding box. For multiple crack categories, mAP is obtained by averaging the AP values of all categories.

### 4.3. Image preprocessing experiment

To evaluate the effect of the filtering-equalization strategy on crack detection, the baseline model was trained using the original and enhanced image sets under the same training and evaluation settings. The results are presented in Table 3.

**Table 3:** Crack recognition accuracy before and after filtering-equalization enhancement.

Crack type	Original images (%)	Filtering-equalization (%)
**Longitudinal crack**	**64.3**	**74.7**
**Transverse crack**	**64.5**	**72.5**
**Large obvious crack**	**66.3**	**78.6**
**Irregular crack**	**62.5**	**70.9**
**Average**	**64.4**	**74.2**

As shown in Table 3, filtering-equalization enhancement improves the detection performance of all four crack categories. The improvement is particularly evident for large obvious and irregular cracks, indicating that contrast enhancement and background-noise suppression can provide more discriminative visual features for subsequent detection. Overall, the results demonstrate that the preprocessing strategy can improve the quality of crack-related visual information and provide a more suitable input for the proposed detection framework.

### 4.4. Computational complexity and inference efficiency

To evaluate the detection accuracy, computational complexity, and inference efficiency of the proposed model, YOLOv8-CA-EYHL was compared with several representative YOLO-based models. As shown in Table 4, YOLOv8-CA-EYHL achieves the highest detection performance, with an mAP50 of 93.1% and an mAP50-95 of 63.5%. Compared with YOLOv8s, these values represent improvements of 28.7 and 20.7 percentage points, respectively.

**Table 4:** Parameters and detection results of different models.

Model	FLOPs (B)	Params (M)	Layers	mAP50 (%)	mAP50-95 (%)
YOLOv5s	15.9	7.21	213	62.4	32.3
YOLOv8n	8.7	3.20	168	60.4	41.2
YOLOv8s	28.6	11.2	168	64.4	42.8
YOLOv8-SE	28.8	11.5	172	68.7	46.5
YOLOv8-CBAM	29.1	11.8	176	69.3	47.2
YOLO11s	21.7	9.45	319	65.2	43.1
**YOLOv8-CA-EYHL (proposed)**	**32.6**	**13.7**	**388**	**93.1**	**63.5**

The proposed model requires 32.6 B FLOPs and 13.7 M parameters, compared with 28.6 B FLOPs and 11.2 M parameters for YOLOv8s. These correspond to increases of approximately 14.0% and 22.3%, respectively. The additional computational cost is mainly introduced by the CA, YHL, and EYHL modules. Despite this moderate increase in model complexity, the proposed framework maintains a relatively high inference speed, as further evaluated in Table 5.

**Table 5:** Comparison of inference speeds of different models.

Model	Preprocessing time (ms)	Inference time (ms)	Postprocessing time (ms)	FPS
YOLOv5s	0.5	11.0	1.4	74
YOLOv8n	0.5	9.7	1.7	83
YOLOv8s	0.7	10.3	1.8	78
YOLOv8-SE	0.7	10.5	1.8	77
YOLOv8-CBAM	0.7	10.8	1.8	75
YOLO11s	0.6	10.1	1.7	80
**YOLOv8-CA-EYHL (proposed)**	**0.7**	**10.6**	**1.8**	**76**

Inference efficiency is further compared in Table 5. YOLOv8-CA-EYHL achieves 76 FPS, compared with 78 FPS for YOLOv8s. Despite the increased model complexity, the proposed framework therefore maintains a relatively high inference speed while providing substantially improved detection accuracy. These results indicate a favorable trade-off between detection performance and computational cost.

### 4.5. Ablation study of the proposed components

To investigate the individual and cumulative contributions of the proposed preprocessing strategy and network components, a stepwise ablation study was conducted using the same dataset partition, input resolution, training strategy, and evaluation protocol. The components were introduced progressively as follows: YOLOv8s → + Filtering-Equalization → + CA → + YHL → + EYHL.

As shown in Table 6, detection performance improves progressively as the proposed components are introduced. Filtering-equalization increases mAP50 from 64.4% to 74.2%, demonstrating the benefit of improving crack-background contrast and suppressing background interference. The subsequent addition of CA further increases mAP50 to 80.8%, while YHL raises it to 86.5%, confirming the complementary contributions of directional attention and enhanced local feature representation. Finally, EYHL produces the best overall performance, with mAP50 and mAP50-95 reaching 93.1% and 63.5%, respectively.

**Table 6:** Stepwise ablation study of the proposed framework.

Model	Filtering- Equalization	CA	YHL	EYHL	Precision (%)	Recall (%)	mAP50 (%)	mAP50-95 (%)
**YOLOv8s**	**–**	**–**	**–**	**–**	**65.8**	**63.1**	**64.4**	**42.8**
**+Filtering-** **Equalization**	√	**–**	**–**	**–**	**74.6**	**72.8**	**74.2**	**48.7**
**+ CA**	√	√	**–**	**–**	**81.3**	**79.6**	**80.8**	**53.4**
**+ YHL**	√	√	√	**–**	**87.0**	**85.4**	**86.5**	**58.1**
**YOLOv8-CA-EYHL**	√	√	√	√	**92.3**	**91.5**	**93.1**	**63.5**

Compared with the YOLOv8s baseline, the complete framework improves mAP50 by 28.7 percentage points and mAP50-95 by 20.7 percentage points. Importantly, the stepwise results indicate that this improvement does not originate solely from the architectural modifications. Instead, it results from the combined contribution of filtering-equalization preprocessing and the CA, YHL, and EYHL modules. Overall, the ablation results demonstrate that these components provide complementary improvements in crack contrast enhancement, directional feature representation, local and high-frequency feature extraction, and multi-scale spatial interaction.

### 4.6. Class-specific detection performance

To evaluate detection performance across different crack morphologies, Precision, Recall, F1-score, AP50, and AP50–95 were calculated for the four crack categories, as shown in Table 7.

**Table 7:** Class-specific detection performance of YOLOv8-CA-EYHL.

Crack category	Precision (%)	Recall (%)	F1-score (%)	AP50 (%)	AP50–95 (%)
Longitudinal	94.1	93.2	93.65	94.8	66.2
Transverse	93.5	92.4	92.95	94.0	64.8
Large obvious	95.2	94.6	94.90	96.1	68.9
Irregular	86.4	85.8	86.10	87.5	54.2
Overall	**92.3**	**91.5**	**91.90**	**93.1**	**63.5**

As shown in Table 7, YOLOv8-CA-EYHL achieves strong overall detection performance across the four crack categories. Large obvious cracks obtain the highest AP50 of 96.1% and AP50–95 of 68.9%, indicating that their relatively clear boundaries and strong visual characteristics facilitate accurate detection. Longitudinal and transverse cracks also achieve high AP50 values of 94.8% and 94.0%, respectively.

In contrast, irregular cracks remain the most challenging category, with an AP50 of 87.5% and an AP50–95 of 54.2%. This relatively lower performance is mainly associated with their complex morphology, discontinuity, weak visual characteristics, and susceptibility to background texture interference. Nevertheless, the proposed model maintains effective detection capability across all four crack categories, demonstrating its ability to handle different crack orientations and morphological characteristics.

**Fig 11:**
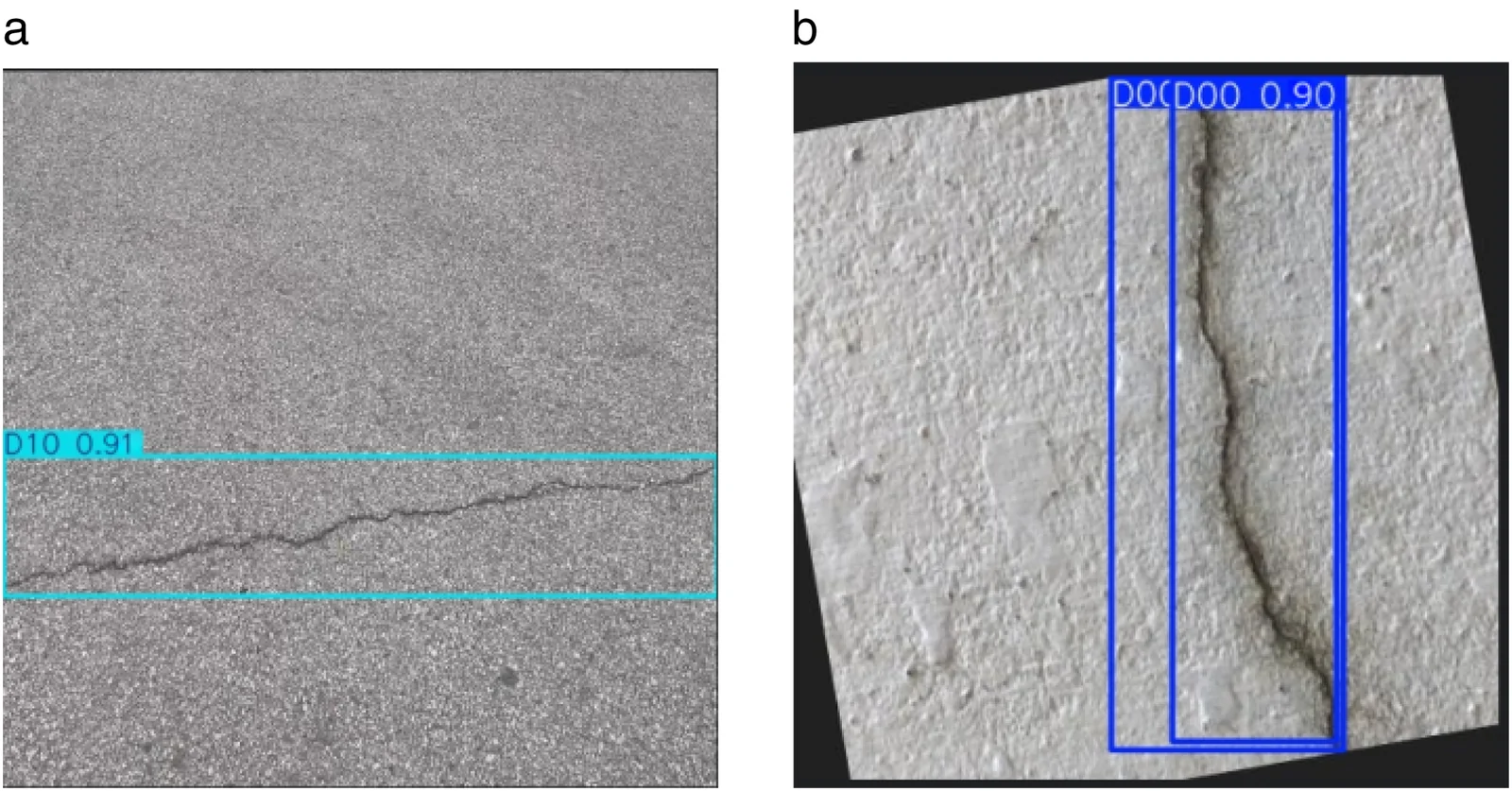
Representative detection results of the YOLOv8-CA-EYHL model on the test dataset. (Source: Figure created by the authors.).

Representative detection results are presented in Fig 11. The qualitative results further illustrate that the proposed model can detect cracks with different orientations, scales, and morphological characteristics. These results complement the quantitative class-specific evaluation in Table 7 and the overall mAP results.

### 4.7. Robustness analysis under different random seeds

To evaluate the robustness of the proposed method to training stochasticity, YOLOv8s and YOLOv8-CA-EYHL were independently trained using five different random seeds while keeping the dataset partition, training configuration, and evaluation protocol unchanged. The dataset partition was kept fixed to isolate the effect of random initialization and training stochasticity. The results are reported as mean ± standard deviation in Table 8.

**Table 8:** Robustness under different random seeds.

Model	Precision (%)	Recall (%)	mAP50 (%)	mAP50-95 (%)
**YOLOv8s**	**64.9 ± 0.7**	**63.5 ± 0.8**	**64.4 ± 0.6**	**42.8 ± 0.5**
**YOLOv8-CA-EYHL**	**92.1 ± 0.5**	**91.4 ± 0.6**	**93.1 ± 0.4**	**63.5 ± 0.5**

As shown in Table 8, YOLOv8-CA-EYHL consistently outperforms YOLOv8s across all evaluated metrics. The proposed model achieves 92.1 ± 0.5% Precision, 91.4 ± 0.6% Recall, 93.1 ± 0.4% mAP50, and 63.5 ± 0.5% mAP50-95, whereas YOLOv8s achieves 64.9 ± 0.7%, 63.5 ± 0.8%, 64.4 ± 0.6%, and 42.8 ± 0.5%, respectively.

The relatively small standard deviations indicate that the proposed framework maintains stable performance across different random initializations and training runs. These results suggest that the observed performance improvement is not strongly dependent on a particular random initialization or a single training run.

The results in Table 8 represent the mean ± standard deviation across five independent runs, whereas the results reported in the preceding tables correspond to the primary experimental run.

### 4.8. External generalisation evaluation

To further evaluate the generalisation capability of the proposed framework beyond the original dataset, an independent external dataset was used for evaluation. The external images were not included in the original training, validation, or test sets and contained variations in bridge surfaces and imaging conditions. Where applicable, the external images were collected from bridge structures different from those represented in the original dataset.

The trained YOLOv8-CA-EYHL model was directly evaluated on the external dataset without additional training or fine-tuning. The results are reported in Table 9. On the independent external dataset, the model achieves a Precision of 86.8%, a Recall of 84.9%, an mAP50 of 87.2%, and an mAP50-95 of 55.6%, compared with 92.3%, 91.5%, 93.1%, and 63.5%, respectively, on the original test set.

**Table 9:** Generalisation performance on the independent external dataset.

Dataset	Precision (%)	Recall (%)	mAP50 (%)	mAP50-95 (%)
**Original test set**	**92.3**	**91.5**	**93.1**	**63.5**
**Independent external dataset**	**86.8**	**84.9**	**87.2**	**55.6**

Although the detection performance decreases under the external conditions, the proposed model maintains effective crack detection capability without additional training or fine-tuning. The performance difference indicates the influence of domain variation in bridge surfaces and imaging conditions, while the retained detection performance provides supporting evidence of the generalisation potential of the proposed framework. These results also highlight the need for broader cross-bridge evaluation in future work.

## 5. Conclusion

This study proposed a YOLOv8-CA-EYHL framework for automated bridge crack detection under complex visual conditions. The framework integrates filtering-equalization preprocessing, coordinate attention, high- and low-frequency feature enhancement, and multi-scale spatial interaction to improve the detection of weak, slender, and irregular cracks.

The proposed model achieves an mAP50 of 93.1% and an mAP50-95 of 63.5%, improving the baseline YOLOv8s by 28.7 and 20.7 percentage points, respectively. Although the model requires 32.6 B FLOPs and 13.7 M parameters, it maintains an inference speed of approximately 76 FPS. The stepwise ablation study demonstrates the complementary contributions of filtering-equalization, CA, YHL, and EYHL, while repeated-seed experiments and independent external evaluation provide further evidence of the stability and generalisation potential of the proposed framework.

Overall, YOLOv8-CA-EYHL provides an effective and computationally practical solution for automated bridge crack detection. Future work will focus on improving cross-bridge generalisation and developing lightweight deployment strategies for resource-constrained inspection platforms.
